# An examination of advanced cancer caregivers’ support provided by staff interventions at hospices in Argentina

**DOI:** 10.3332/ecancer.2012.281

**Published:** 2012-11-27

**Authors:** Natalia Luxardo, Eugenia Brage, Cynthia Alvarado

**Affiliations:** 1 National Council of Scientific and Technical Research (CONICET), University of Buenos Aires (UBA), Buenos Aires, Argentina; 2 University of Buenos Aires (UBA), Buenos Aires, Argentina; 3 Hospice San Camilo, Olivos, Buenos Aires, Argentina

**Keywords:** family caregiver, end-of-life care, hospice support

## Abstract

The aim of the study was to describe the type of intervention provided by hospice staff in order to address the pragmatic, psycho-social, and spiritual needs of home-caregivers for patients in the last stage of cancer. The qualitative inquiry was carried out in real life contexts. The explicit demands that caregivers (*n* = 40) identified in the first interviews were: (1) helping to organize the care of the patient at home; (2) unspecific demands, with unclear or unrealistic purposes (e.g., curative treatment or a miracle expected to occur); (3) specific resources (such as formal caregivers to replace them), and (4) a place to leave the patient either for a temporary period (a respite for the family) or in a permanent way. The main issues discussed were the delays in the patients’ referral to the hospice and the lack of time for long-term interventions; explicit focus is placed on the care by addressing the spiritual and emotional needs of caregivers, unlike in hospital settings where professionals avoid discussions of spiritual needs due to a lack of time, inadequate training and poor understanding of spirituality; hospices’ interventions are based upon an ethos similar to the movement’s original Christian spirit with emphasis placed on qualities of care such as love, charity, and compassion besides expertise and end-of-life competence, all while tolerating a sense of abandonment by health and social security systems following the patient’s referral.

## Introduction

The provision of home-care for a dying relative can be a positive and rewarding experience because it may reduce feelings of guilt, provide a purpose in life, fulfil a sense of duty, and help in coping with bereavement, among other factors [1]. However, if the care situation is extended in time and without optimum support, the impact upon the caregiver’s health status can be negative [2, 3]. Many scholars [4–7] have identified various stressors to informal caregivers which negatively affect their physical, psychological, and emotional health. Therefore, the support provided for caregivers by specialists is a decisive matter in end-of-life issues. The concept of support is used in relation to family caregivers and is considered desirable. However, an understanding of being or not being supported remains unexplored and not comprehensively described [8]. Docherty et al.’s [9] review identified that in palliative settings there was weak evidence for caregiver’s information needs in relation to welfare and social support. Research associated with family caregivers has shown differences between the care provided within the context of palliative and hospice care, and the care provided in hospitals [10]. Limited knowledge is available regarding different types of interventions that should be proposed in meeting the needs of family caregivers [11]. As many authors argue [12], the knowledge of what caregivers expect from the home-care team is often lacking. Also the analysis of the dynamics of the relationship between caregivers and staff seems to be infrequent, in spite of the fact that many studies have insisted on addressing the importance of informal caregivers of feeling visible in the healthcare system (such as healthcare professionals’ assessment) and their needs detected [13].

The aim of this study was to describe the support for family caregivers who care for a dying relative at home receiving hospice care. This research was specifically based on a type of intervention provided by the hospice to address the pragmatic, psychosocial/educational, and spiritual/emotional needs of caregivers for advanced cancer patients in the last stage of the disease.

## Methods

The design was a qualitative case study. The empirical inquiry was made within real life contexts: either at the hospice—House of Hope [*Casa de la Esperanza*]—or the caregivers’ homes. The ethnographic fieldwork in both scenarios was carried out in 2010–2011. The design also combined grounded theory procedures, specifically for the analysis of data. Two separate samples were taken, either with staff or caregivers. Both were small, non-random and convenience samples. With regard to the staff sample, among the 80 volunteers that work in the three programmes that the hospice offered (“Hospice at Hospitals,” “Hospice at *Casa de la Esperanza*,” and “Hospice at Home”) were selected, we chose only those people involved in “Hospice at Home,” since it was part of the wider research carried out by the main researcher (NL) in palliative care services with a focus on home-caregiving. The “Hospice at Home” staff was made up of a nurse, two psychologists, a physician, a priest, and volunteers (up to five, but not all of them working at the same time). The caregivers’ samples were chosen by examining the forms of the hospice’s registry of 400 patients cared for from 2002 to 2011. We selected 40 patients, according to the following criteria: participation in the programme “Hospice at Home,” having family caregivers as the main resources for their care (vs. formal caregivers), and the possibility of re-establishing contact with the caregiver when the their situation had finished. From these, we selected nine as informants (more precise details about this second selection will follow).

For the data collection, we relied on three main sources. Firstly, field journals that registered direct observations of the daily dynamic of the hospice (emergent’s dimensions) in order to explore how categories for admissions were established. Secondly, the participant observation of the staff in weekly meetings to organize the interventions with the focus on patients being cared for at home. The meetings were audio-taped and later transcribed to a matrix with the dimensions of the study being previously determined. Thirdly, semi-structured and/ or open interviews conducted in the patient’s home (five) and in some cases these were tape-recorded interviews. There were also four ex-caregivers included, that had cared for patients within six months, who were now deceased. It was decided not to extend the period of recruitment due to the loss of contact between families and the hospice as time went by. The established selection criteria were based on three conditions. Those chosen needed to have belonged to the programme, to have been the primary caregiver, and to have a willingness to participate in the interviews. As a secondary source of data, the biographical forms filled by the staff over the last year, regarding interventions with patients and families’ assessment were included. Their main focus was related to initial contact, explicit demands and the history of how the interventions were implemented and discussed within the staff.

The hermeneutic approach was used to analyze the data, combining analytic techniques from grounded theory and narrative analysis in a flexible manner. Firstly, data was analyzed through repeated readings and listening to audiotapes of the staff meetings and the initial interviews. They were then transcribed into written text using modified open coding. We examined the data and identified components of caregivers’ responses and attitudes to homecare. Secondly, using axial coding [14], we organized and connected the dimensions of the data, making comparisons between them and identifying similarities and differences. This form of constant comparative analysis was used to generate three main emerging themes: the initial contact; the different kinds of support provided and the limits in addressing other needs in caregiving. Two ways of validating our findings were implemented. We triangulated this data with an analysis of the secondary sources, which gave supplementary information, but not new categories. One of the researchers (CA) was a nurse on the hospice’s staff, significant comprehension of the data was possible thanks to her firsthand perspective. The coding was discussed in focus groups with the rest of the staff, who enhanced the reflexivity in the analysis by providing new insights into the dimensions selected and were permitted deconstruct only theoretical assumptions and root them in daily experiences.

In regards to ethics, approval for this research was obtained from the Hospice’s Committee of Directors. It was observed that there was a risk of intrusiveness and distress by including the interviews of bereaved caregivers. However, we could justify our decision properly based on Seamark et al.’s [15] findings. They demonstrated that over 80% of those interviewed showed only mild or no distress, and it could even have therapeutic results in this context. They concluded that post-bereavement interviews with primary caregivers could be successfully conducted without undue distress. We also relied upon the follow-up bereavement support and strategies that the hospice provides for caregivers with unresolved grief. This includes weekly staff phone calls to the bereaved during the first month; home visits, if necessary; and the invitation to share the Low Mass offered by the hospice religious personnel once a year to commemorate relatives that had passed away. Verbal consent was obtained from the staff, and the caregivers were interviewed, after explaining the purpose, methods, and audience for the final results and the well-known issues in ethical protocols: being anonymous, the freedom to withdraw at any moment during the study, the possibility to access all the results and having an available contact person in the research team. All the raw data was protected as confidential.

## Results

### Socio-demographic data

We included 40 families, in which 70% (28/40) of the main caregivers were women, and 75% (30/40) were either a spouse or a daughter. The subjects were from the lower middle and lower classes. Since no questions about incomes were used in the hospice’s forms, two proxy indicators were considered for this classification: the ability to pay for medication and the type of medical insurance of the patient. Also, 70% of the principal carers resided with the patients. Most of them lived in Buenos Aires.

### Initial contacts

Most of the caregivers made contact with the hospice by responding to a friend’s or acquaintance’s suggestion (35/40), a small proportion came from institutions (2/40) and it was rare that patients instigated the initial contact with the hospice themselves (1/40). This data shows the lack of institutional network and the isolation of the hospice, despite the necessary initiatives to be implemented in order to be known. The admission criterion was quite flexible when compared with formal institutions. A special team was in charge of either accepting or rejecting the family’s request, based upon the three following priorities: the disease being in a terminal phase, low income and lack of/or poor family support. The explicit demands that caregivers identified in the first interviews were: to help to organize the care of the patient at home (26/40); unspecific demands, with unclear or unrealistic purposes—e.g., curative treatments—(7/40); specific resources, such as formal caregivers to replace them (3/40) and a place to leave the patient either for a temporal period—a respite—or permanently (2/40).

With regard to the initial approach, the staff adopted a respectful and cautious attitude to the atmosphere of the home, checking that they were not disturbing the patient’s intimacy or what was expected of them. They entered patients’ homes while aware of the risk of transgressing the borders of privacy and intimacy and tried to avoid doing so.

One of the first evaluations they carried out was related to the degree of information that families and patients had about diagnosis and prognosis. Conflicts arose when patients and caregivers had different perspectives and attitudes regarding open-awareness contexts and expectations about daily care. In many cases, caregivers and the patient’s needs and wishes were not only different but opposites. In the interviews, some caregivers expressed the expectation of hospitalizing the patient during their last moments, because they did not feel ready to deal with the agony of the loved one. However, in examining the field journals it was observed that the patient’s wish to *die at home* was a very common request. The staff carefully evaluated both patients’ and informal caregivers’ knowledge about the lack of curative treatment, the need for palliative interventions and prognosis. We detected that 24/40 patients knew of these matters, whereas 4/40 did not. It was remarkable that for 12/40 there were either no data or caregivers preferred not to speak about these key factors. Most of the families were unable to speak honestly with the patient about what was going on, despite the fact that—as noted—the majority of them were aware of both the diagnosis and the prognosis. This was the first main topic in the interventions: to promote a clear and honest dialogue within the family and to carry out visibly what everybody already knew was going to happen. Communication among caregivers, patients and the other members of the family was encouraged and promoted by staff intervention. In doing so, the staff considered that effective dialogue meant speaking to caregivers and to patients in a clear language that was understandable to them, permitting doubts and fears to be expressed and relevant questions asked.

A caring ethos was adopted, based upon the original Christian inspiration of the hospice movement. Thus, the interventions were permeated by a “humble” approach, and an awareness of their own limitations. Phrases such as “lovely approach,” “careful advice,” and “compassionate attitudes” were registered during fieldwork, intimating that such attributes were key components which were as important as competence.

The second important issue was to assure families that they were not alone anymore, which implicated being close and attentive to the needs of the family’s caregivers. Being supportive in terms of staff perspective meant *being there* or, at least, being available for the family’s needs. Our data showed that in 39/40 interventions, personal phone numbers were given to the caregiver. In the interviews, the caregivers often expressed feelings of being supported by telephone-based counselling, and appreciating its availability and the immediacy of contact, in contrast with bureaucracy of the hospitals.

### Different types of support offered

The focus of interventions included pragmatic or instrumental support [16], psycho-educational and social support, spiritual and emotional support, caregiver coping, symptom management, sleep promotion, family meetings and bereavement support. Only the three first factors will be described here. As regards pragmatic support, one of the main difficulties for the family caregivers was to combine the demands of the care situation with ordinary daily activities such as housework, shopping, maintaining the home, outside commitments, and childcare. As a result, pragmatic support for being able to deal with such things was the concern that caregivers most frequently expressed. By examining the interviews and field journals, we observed that the principal practical arrangements that needed to be addressed were: intimate bodily care, dealing with incontinence, providing care for a wholly dependent person, preventing pressure sores, dealing with specific recipes that required homemade meals and monitoring the patient’s signs and symptoms over 24 h per day. The staff evaluated the available resources, trying to avoid a situation in which all the patient care demands fell upon a single person, the primary caregiver. A strange but not uncommon situation was when the interventions identified a *burnt out* caregiver, or even a kind of “fake” caregiver; someone performing a role just because of circumstances and being unable to express the discomfort of doing so. These occurred when a previously poor relationship existed between them. There were caregivers that, due to persistent complaints, continuous state of overexcitement, and the high levels of anxiety expressed, only made the care situation worse; while also impeding others in providing the proper care that they were unable to give. In such cases, caregivers felt forced by social pressure to continue with this *façade,* and as one put it: “he doesn’t deserve all this caring, he was a violent husband and an absent father… I’ll stand by him, but nobody can expect that I could turn into a devoted nurse”. Being supportive for the staff consisted of facilitating the family caregiver in terms of providing opportunities to relinquish care and include others in the process. Allowing the family caregiver to express her or his feelings and thoughts with regard to the care and the patient in a proper context entailed new strategies to cope with the care situation, rather than merely rejecting it as the sole option. Also, the staff promoted the reflection on and open discussion about the meaning of care as a way to enrich the relationship among family caregivers and the patient, identifying the achievements during this period of care in order to avoid future remorse. By finding meaning in caring and satisfaction and by avoiding specific tasks caregivers found unbearable to perform—such as intimacy toileting when the caregivers and patients belong to different generations—were very important coping resources. Remorse and feelings of guilt or failure were not found among the four caregivers’ post-death narratives. On the contrary, they highlighted a sense of profound pride at having enabled the loved one to die in their own environment and in a dignified manner.

The caregivers expected the hospice members to have specialised knowledge of end-of-life care and they demanded this *expertise*. The staff reassured caregivers of the feasibility of providing care at home until the end, teaching the family different ways to handle situations that made caregivers feel in control with a sense of security and safety related to homecare. In the field journals, there were many examples of caregivers’ surprised questions after conversations with a staff member, such as: “…so you are saying that dying at home is possible?” Some caregivers also expected the provision of respites, because often they felt overwhelmed by the activities they had to perform for patients 24 hours per day. So, many of them took respites informally, during staff home visits, when they left the house for some hours. Making a request for admittance of the patient at the hospice’s residence was also a common strategy.

The staff were also involved as liasons between formal resources and caregivers. They had strategies to help the family get access to social benefits. This was a very important issue to address, since in the economic situation of the families the level of hospice care was low, especially during disease trajectories. We also identified households of patients who had had to take on a reduction in hours of work which had caused a loss of family income and delays in paying for essential services—with the danger that these services might be interrupted, properties sold, savings used up, and families needing to take out loans and use credit. In general terms, some were unable to work in either paid or formal employment. Caregivers reported that finances were strained not only by the reduced income but also by the increased family expenses related to the disease.

The second type of support was psychosocial-educational. Among the most important goals of hospice care was the provision of psycho-educational support for patients and families facing terminal illness. One of the areas we identified within this dimension was related to symptom management and medication administration. Often the caregiver could not understand the exact type of suffering that the patient had been experiencing. There were caregivers that did not give patients any medication at all despite the pain they were in, because they thought that the complaints and the suffering were only related to “psychological distress,” or because they considered suffering as something “natural” and unavoidable in the terminal stage, as one man explained: “A. [his wife] has the worst of the cancers, so I told her, nothing can be done [to alleviate her pain] but to resign and to put yourself in God’s hands.”

Conversely, sometimes, carers of family members being treated with medication had only feelings or expressions of anguish, so they asked for help to make the patient sleep all day because they could not stand listening to them crying, complaining, or insulting them; thus overmedicalizing the patient’s own reactions to death. Relatives in such cases did not always fully consider the uniqueness of the death experience, which may be expressed in the most heterogeneous modalities, not necessarily in a peaceful and quiet manner. The nurse wrote on the hospice’s form: “We explained to D. that G.’s sedation can’t be her anxiolytic. If she isn’t prepared to withstand this moment, she should think of some other ways of coping, such as visiting a psychologist”.

The hospice staff found a medication strategy necessary to provide assistance to those who had this ambiguous concern about the patient’s pain. Firstly, they identified which type of pain it was, and when all the technical and proper measures were taken, they explained to the caregivers that the patient’s complaints might be related to a personal reaction toward their own death. Therefore, the focus was on respecting the method the patient used to express feelings about the end of life, either through tears, insults, yells, words, or whatever, avoiding palliative sedation. A special case we identified was related to changes in the morphine prescriptions that caregivers usually had to administer. Even when nurses recommended a schedule, caregivers often did not respect it, with excuses such as “What for? She is not in pain now”, “I was about to give him the pill, but I was ‘stretching the time’ as long as possible, because if we begin with high doses now, we might not have anything to calm him later”. Some of these inadequacies in caregivers’ knowledge of pain management had to do with prejudices and myths related to opioids and pain killers in general. For instance, the fear of the use of morphine was common, with comments such as “he will become an addict”, “the secondary effects are worse than her pain”, “we don’t want drugs in our house”, “that’s the medication of last resort, for someone that will die within the next few hours, not for her”. Counselling for pain avoidance was reported in 22/40 interventions and counselling for symptom management 30/40.

Another crucial dimension within this kind of support had to do with working for the preparedness caregivers needed for coming to terms with what is going to happen. Staff put the emphasis on showing what was expected to occur, what was not, and what important issues needed to be discussed in advance in order to avoid decisions being made in the immediacy of the emergency. Many caregivers expressed the feeling of living in uncertainty and in a state of complete unpredictability with regards to the patient’s constant physical, emotional, and cognitive changes. The staff’s psycho-educational interventions provided a basis for realistic and anticipated expectations and avoided caregivers being lulled into false hopes, so that the caregiver’s anxiety and fears could be reduced. Through the staff’s normalizing of the situation, family caregivers were—in a way—ready to accept or at least to recognize what was going to happen. Many caregivers seemed not to perceive that the cared for person was in the process of dying.

In post-bereavement interviews, we detected a shift in the perception of carers. As the weeks and months went by, new perspectives arose based upon the evaluation of how things had finally turned out. For example, they expressed that interventions focused on anticipatory events and decisions had been valuable, even when they might have been reluctant to accept them when they were offered, because they felt they were being pushed into events and decisions before they occurred. This type of intervention was noticeable in almost all of the hospice approaches (37/40), more than practical or emotional support.

Finally, emotional and spiritual support—salient issues in the hospice’s interventions—will be described. The spiritual dimension was analyzed by considering spirituality as the universal search for meaning, values, and purposes, and in identifying which were the specific spiritual needs in caregivers’ narratives and how they perceived that the hospice staff might be supported in addressing these issues [17]. However, we also considered that one important belief system that is commonly relied upon during times of uncertainty (including caregiving) was religion [18]. The hospice staff had much expertise in dealing with the spiritual dimensions, an ordinary attitude rooted in the original Christian spirit of the hospice. Most of the staff were explicitly Catholic, thus they were good at facilitating and dealing with resources that belonged to this religion when they were requested by patients or caregivers. This included attendance at formal religious services, the anointing of the sick, confession, home-communion, etc. They also deliberately talked about these topics with patients, based on the conviction that spiritual beliefs help patients to make sense of disease and death. The caregivers’ spirituality was also addressed by promoting reflections about the balance of the caregiver’s life, the forgiveness of some attitudes patients might have held against them previously, and issues of legacies, etc.

With regard to religious help to establish the meaning and purpose of what was going on, caregivers put it clearly: “God knows why things happened”, “We are in His hands, nothing depends on ourselves”, “This helps the family to be closer. Our faith in God made us stronger”, “All my sacrifice [caregiving] will be rewarded someday”. Being stronger, being not responsible for—and thus, a freedom to be in charge or in control—and finding meanings and answers were some of the beneficial consequences we found in our research regarding religion and well-being among caregivers. Many caregivers and patients mentioned that even when they did not believe in God, they found being a witness to Catholic rituals peaceful and comforting. Some caregivers refused this type of approach, not always being able to express themselves explicitly and being unable to criticise the staff’s good intentions.

However, the needs of the broad range of belief systems from the ethnically diverse population of Argentina were not always properly addressed. The hospice was open to all religions. Examining the religion of patients and caregivers, we found that 16/40 were Catholics, 2/40 Jewish, 2/40 Protestants, 2/40 atheist but that the majority (18/40) did not answer the question about this. To explain the refusal to answer this it may be considered that, despite this inclusive perspective, traditional beliefs were less likely to be understood, and some staff members still maintained a belief in certain stereotypes regarding these culturally distant groups. For instance, the attitudes of traditional healers, *curanderos*, who proposed other ways of coping with disease, were considered something to change. An association of lower economic status and educational levels with these kinds of beliefs was sometimes made by staff members during the interviews. So, as economic changes could not be proposed as an intervention, the variable that was possible to affect was the second one: reinforcing education. There was a lack of cultural, historical, ethnic or contextual clues to understand caregivers’ and patients’ traditional practices.

The emotional support interventions were also a distinctive and explicit goal of the hospice’s staff. Some caregivers felt ashamed of asking to be replaced by others because they wanted to laugh, to relax, and to have fun when the loved one was about to die. Caregivers knew that certain licenses were permitted and were socially and morally considered good and reasonable (such as job duties, physical health impairments, etc.) whereas others were condemned (e.g., hobbies). These social expectations often turned into a self-pressure to accomplish what society considered an obligation. In this regard, the staff supported caregivers’ feelings and validated their wishes and preferences, without judging them. Being supportive in this way often simply meant being able to validate the caregivers’ wishes, make them clear, and encourage caregivers to pursue them.

The nature of this support and the form it took differed also from palliative teams and from other clinical services. The hospice staff was not only explicitly religious but also explicitly connected with *caring emotionally*, which implied providing care with love and compassion, not just technical competence. Touching, hugging, kissing, holding hands, caressing, and all expressions of connection and interest in the other were used, either with family caregivers or with patients.

### Hospice’s limits

The average time that the hospice spent with each patient was 37 days. There was an overall range from a couple of hours to almost a year in the hospice. We noticed that even when the sudden death of the patient was unexpected, at other times the decision to go to the hospice at the—almost literally—last hour of life was an agreement made among patients and their families, a conscious choice in order to be able to pass away without feeling the pressure of the loved ones to remain “alive at all costs.” The hospice represented the selected peaceful geography that, within a religious context, could help relatives during this last sad moment.

This delay in the caregivers and patients hospice contact was responsible for the lack of time necessary to treat aspects of that family that required more time to be explored, since confidence generally needs to be established gradually. In connection with this delay, we analysed two caregivers’ interviews in which the staff could not recognize that in intervening they were imposing external elements onto the patient-caregiver’s world. When staff talked openly about death and the short time of life the patient had, or the lack of curative treatments in front of people who considered it important to remain hopeful of recovery, and even when caregivers remained silent, some sort of disapproval for that open-awareness context was perceived. In such cases, the avoidance of the open acknowledgement of death was not respected since for many social and ethnic groups, hope was more important than truth-telling. There was an implicit perspective that hospice enrolment meant that the patient was about to die. However, for many patients and their families, this was not something obvious or evident. It did not matter that, with the information given by physicians and staff, they still had hope in recovery, or at least, they wanted to have it. Some caregivers were surprised and disturbed by this open communication which they found to be harmful to the family. This was a great dilemma, because the staff recognized that most of the times patients did want to know, and already knew, so keeping what was called a ‘conspiracy of silence’ only made the whole situation worse.

## Discussion

In the hospice’s interventions, an ethos similar to the original spirit of the hospice movement remained [19], with a focus on values such as love, charity, and compassion, as well as expertise and end-of-life care competence. The staff had an explicit goal of caring by addressing the spiritual and emotional needs of caregivers, contrary to hospital settings where professionals often avoided discussions on this topic due to a lack of time, inadequate training and a poor understanding of spirituality [20]. On the contrary, the hospice’s staff had extensive training in addressing these needs, and many authors have observed caregivers coping better with adversity [18]. The emotional support most commonly given consisted of simply to listening to and validating caregivers’ feelings, and encouraging them to enroll in leisure activities and reserve a time for enjoyment and relaxation with others, avoiding isolation. The lack of social support for the caregiver could account for the breakdown in care.

However, we also identified a mismatch between the staff caregiver’s assessment of the burden and that of the primary caregiver with regard to spiritual needs. Specifically when the primary caregiver asked for instrumental and not spiritual support, leaving them to feel a subtle pressure to accept the other due to the religious characteristics of the institution. The findings showed that caregivers usually and explicitly required more help with the activities of daily living and domestic chores, thus practical support and psycho-educational interventions strengthened their ability to cope with end-of-life crises.

As Kenny et al. [21] stands for clinicians caring for palliative care patients, it is important to be alert to the potential physical and psychological impairments of informal caregivers, ensuring that they were adequately supported in their caregiving role with permanent home visits and phone-calls implemented and facilitating access to the appropriate care. One of these types of care was to legitimize the “not (more) ready for caregiving” option.

Cultural factors should be introduced into understanding families’ strategies in illness trajectories. This is necessary to ensure that supportive services are both meaningful and culturally appropriate without external senses being attached. Otherwise a subtle way of coercive moral imposition could unintentionally occur.

## Conclusions

This research was intended to contribute to the literature on caregiving by providing insights into the social, cultural, and spiritual context of informal family caregiving in a hospice program, identifying the pros and cons of the interventions implemented by the hospice staff for better support for home caregivers. As Donovan [22] argues, families are facing increased pressure to provide care to their terminally ill or dying kin in the home. Being able to conciliate personal and social roles with the caregiver role can adversely affect them, especially when access to supportive services is inadequate. From the families’ perspective, receiving services from a range of providers was perceived as being overwhelming and confusing due to the lack of coordination among them, and also because sometimes the indications were contradictory, leaving families in the uncertain situation of choosing between multiple paths. Non-governmental organizations—such as the provision of support in hospices—cannot stand as the only answer. Many authors [23] noticed that within global policies, having patients in their homes and having caring relatives not only implied financial costs but also indicated the existence of an unequal level of access to medical care and resources. We detected throughout the study that caregivers felt isolated and resentful of the lack of support from the formal system, with a sense of abandonment by health and social security systems. Hospices and hospitals could not work jointly due to the fact that the formal system often collapsed under the strain of such demands, so once patients were enrolled into the hospice, contact with health care professionals was less frequent.

The level of perceived support that caregivers expressed with regard to hospice availability was necessary for relieving, partially, what is called the *burden of care*. However, besides supporting family caregivers, it should be stressed that addressing the responsibility of the sanitary system for providing adequate alternatives to deal with end-of-life care at home is necessary. Otherwise, current end-of-life policies will keep maintaining and reproducing social and gender inequity, and delegating to non-governmental organizations what they fail to provide.
